# Examination of the causal role of immune cells in non-alcoholic fatty liver disease by a bidirectional Mendelian randomization study

**DOI:** 10.1515/med-2025-1154

**Published:** 2025-02-19

**Authors:** Yu Li, Xiaodan Lv, Jianing Lin, Shiquan Li, Guangfu Lin, Zhixi Huang, Deyi Chen, Lichun Han, Lingling Zhan, Xiaoping Lv

**Affiliations:** Department of Gastroenterology, The First Affiliated Hospital of Guangxi Medical University, Nanning, 530021, Guangxi, China; Department of Clinical Experimental Medicine, The First Affiliated Hospital of Guangxi Medical University, Nanning, China

**Keywords:** Mendelian randomization, non-alcoholic fatty liver disease, immune cell, causal inference

## Abstract

**Background:**

Non-alcoholic fatty liver disease (NAFLD) is a globally widespread disease. Recent investigations have highlighted a close association between immunity and NAFLD, but the causality between them has not been thoroughly examined.

**Methods:**

A total of 731 immunological traits and NAFLD cohorts were derived from genome-wide association study summary data, and single nucleotide polymorphisms significantly associated with immune traits were identified as instrumental variables. Moreover, 731 phenotypes include absolute cell counts, median fluorescence intensity (MFI), morphological parameters, and relative cell counts. The bidirectional two-sample Mendelian randomization (MR) was performed primarily using the inverse-variance weighted methods, and sensitivity analysis was carried out simultaneously.

**Results:**

Four immunophenotypes were identified to exert a protective effect against NAFLD, including HLA-DR^+^ CD4^+^ %lymphocytes, SSC-A on CD4^+^, CD24 MFI on IgD^−^CD38^−^, and CD8 MFI on CD28^−^CD8^br^. Seven immunophenotypes were identified to be hazardous, including CD28^+^ CD45RA^+^ CD8^dim^%CD8^dim^, CD127 MFI on CD28^+^ DN (CD4^−^CD8^−^), CD20 MFI on IgD^+^ CD38^br^, CD20 MFI on transitional, IgD MFI on transitional, CD3 MFI on central memory CD8^br^, and CD45 MFI on CD33^br^HLA-DR^+^ CD14^−^. However, reverse MR showed NAFLD had no causal effect on immunophenotypes.

**Conclusion:**

The study demonstrated a potential causal link between several immunophenotypes and NAFLD, which contributes to advancing research and treatment of NAFLD based on immune-mediated mechanisms.

## 1 Introduction

In 1980, non-alcoholic fatty liver disease (NAFLD) was first proposed to define conditions with histological features similar to those of alcoholic liver diseases [1]. NAFLD progresses through non-alcoholic fatty liver and non-alcoholic steatohepatitis (NASH), potentially resulting in severe fibrosis and cirrhosis [2]. Although numerous studies have pinpointed effective interventions for specific cirrhosis-related complications [3], there remains a lack of viable therapeutic options for liver fibrosis, irrespective of the underlying etiology, including NAFLD [4]. With a global prevalence of 25%, NAFLD is acknowledged as the primary contributor to chronic liver diseases and cirrhosis, imposing a significant burden on the global economy, especially in the Middle East, Asia, and North Africa [5].

Unfortunately, the prevalence of metabolic risk factors for hepatocellular carcinoma (HCC), like NAFLD, is rising and could eventually become the predominant cause of HCC worldwide [6]. The precise etiology of NAFLD remains unclear. Research indicates that NAFLD development is attributed to metabolism, gut microbiota, immune responses, and environmental elements. A comprehensive understanding of the disease as a complex interplay of various etiological factors based on immune responses will help to refine the current clinical insight into NAFLD and unveil new therapeutic options [7,8]. A study has demonstrated that activation of silent information regulator 1 (SIRT1), a transcription factor associated with the pathogenesis of NAFLD, can significantly repress inflammatory responses during liver injury [9]. Importantly, activation of intestinal lymphocytes and immune responses in the liver is associated with chronic low-grade inflammation, a primary etiology of NAFLD [8].

The inflammatory environment in NASH is predominantly governed by immune cells from the innate and adaptive systems. Immune cells secrete inflammatory mediators to induce hepatocyte death, while stressed hepatocytes are more prone to cytokine-mediated cell death, thus releasing molecular substances known as damage-associated molecular patterns (DAMPs)[10]. Many infiltrated innate immune cells, encompassing neutrophils, monocytes, dendritic cells (DCs), and Kupffer cells, contain pattern recognition receptors (PRRs). DAMPs activate PRRs to induce sterile inflammation through immune responses [11]. Thus, innate immune responses are acknowledged as crucial contributors to NASH development. However, accumulating evidence suggests that adaptive immunity is equally important. It has been reported that liver injury and lobular inflammation are closely associated with the degree of recruitment of CD4^+^ and CD8^+^ T lymphocytes in the methionine choline-deficient model of NASH [12]. Evidence from high fructose-induced models of NAFLD supports that CD8^+^ T cell depletion can protect mice from developing steatosis [13]. However, contrary to previous beliefs that adaptive immunity predominantly facilitates NASH progression, recent research indicates that adaptive immune responses may be a double-edged sword [14,15]. While previous observational articles have unveiled the association between immune cells and NAFLD [16,17], this association may be disrupted by confounders and reverse causality. Additional evidence is warranted to uncover a more robust causal connection. Hence, it is urgent to adopt additional research methods to reveal the causality between immune inflammation and NAFLD, as well as to pinpoint potential treatments.

Mendelian randomization (MR) is a methodological tool that leverages genetic variation as an instrumental variable (IV) to imitate the biological connection between a particular exposure and outcome. Genetic variants such as IVs are allocated from parents to offspring during gamete formation, constituting a form of natural randomization. This approach may minimize the influence of confounders, optimize resource allocation, and avoid reverse causality to a certain degree [13,18]. MR has been applied to infer causal relationships among various diseases [19]. This study employed bidirectional MR to uncover the causal connection of immune cells with NAFLD.

## 2 Materials and methods

### 2.1 Study design

**Figure 1:**
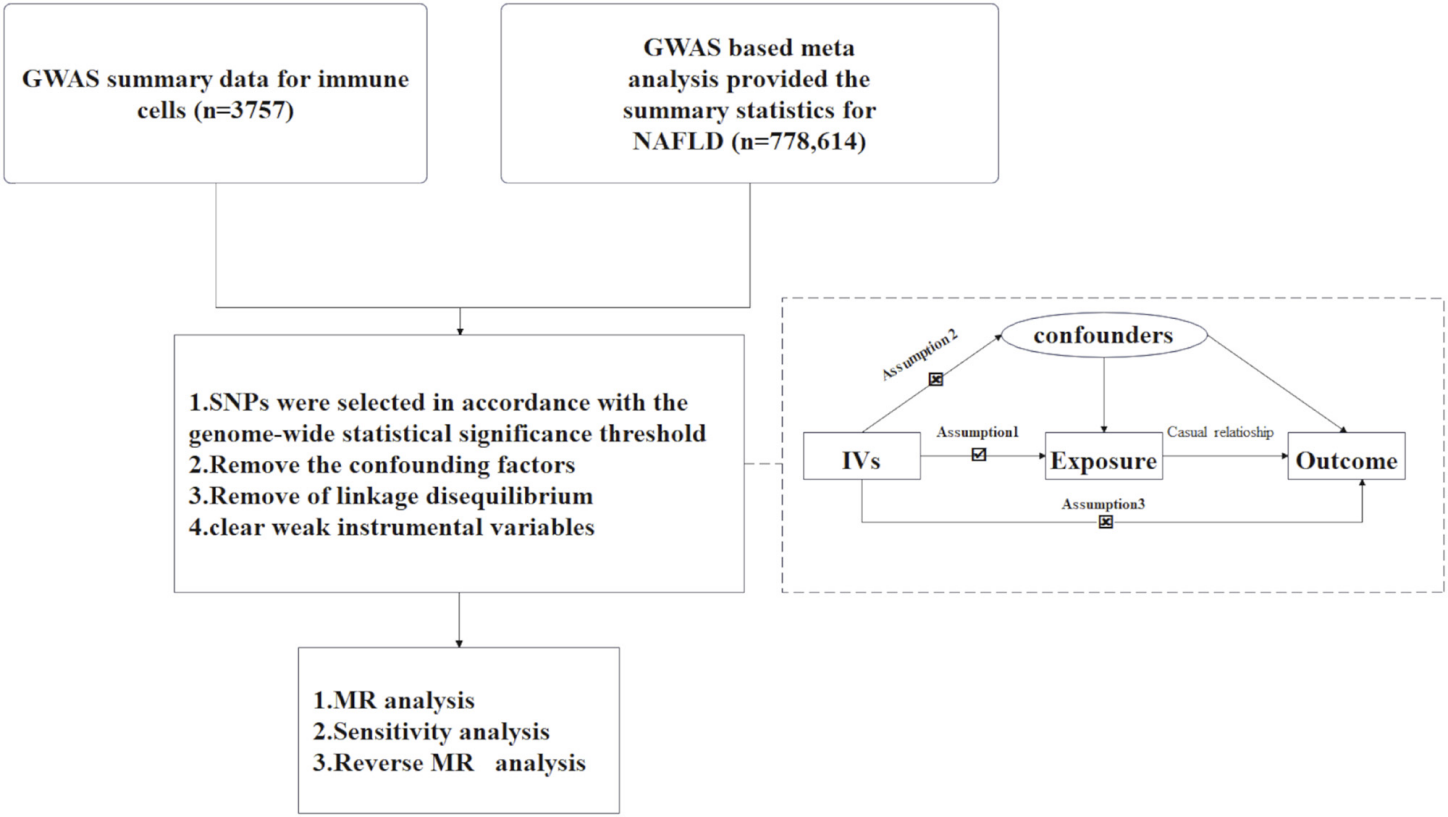
The study flowchart. Assumption 1: IVs have direct connection to the exposure; Assumption 2: IVs are independent of confounders; Assumption 3: IVs influence the outcome exclusively via the exposure; NAFLD, non-alcoholic fatty liver disease; SNPs, Single-nucleotide polymorphisms; MR, Mendelian randomization.

A two-sample MR approach was utilized to determine the causal relationship between 731 immune cell signatures (spanning 7 panels) and NAFLD. The study flowchart is displayed in Figure 1. Genetic variations served as IVs, which required that valid IVs for causal inference satisfied three crucial assumptions: (1) IVs have a direct connection to the exposure; (2) IVs are independent of confounders, meaning that they are not associated with the outcome through confounding pathways; and (3) IVs influence the outcome exclusively via the exposure. All research referenced in the genome-wide association study (GWAS) was authorized by ethical review committees and obtained informed consent from each participant.

### 2.2 GWAS data sources

Publicly available GWAS summary data for various immune traits are accessible from GCST0001391 to GCST0002121 [20]. A total of 731 immunophenotypes in the GWAS catalog were categorized into seven panels: B cells, conventional DCs, maturation stages of T cells, myeloid cells, monocytes, Treg, and T, B, natural killer (TBNK) cells. TBNK panel is a commonly employed immune-monitoring tool that allows for simultaneous detection of T, B, and NK cells. The 731 phenotypes include absolute cell counts (AC, *n* = 118), median fluorescence intensity (MFI, *n* = 389) for surface antigen levels, morphological parameters (MP, *n* = 32), and relative cell counts (RC, *n* = 192). GWAS initially focused on 731 immune traits, leveraging data from 3,757 European samples. Based on 3,757 Sardinian samples, GWAS discerned nearly 22 million single-nucleotide polymorphisms (SNPs) using high-density arrays after adjustment for age, sex, and age^2^ [20,21].

### 2.3 Data source for NAFLD

The largest genome-wide analysis for NAFLD was acquired from 4 cohorts of European participants with health records, encompassing 8,434 cases and 770,180 controls, as well as approximately 6.8 million SNPs [22].

### 2.4 Selection of IVs

Genetic variants were selected from GWAS for IV models according to recent studies [20,23]. Those with a *P*-value less than 1 × 10^−5^ were identified. Then, we eliminated SNPs with notable linkage disequilibrium, defined as *r*² > 0.001 and a distance <10,000 kilobases, to confirm the independence of the screened SNPs. The PhenoScanner database was utilized to examine whether SNPs meet both the independent and exclusion assumptions, while SNPs directly associated with confounders and outcomes were removed [24]. The *F*-value was calculated, and IVs with a value >10 were retained, indicating the absence of weak instrumental bias [25]. Finally, data from both databases were harmonized to ensure that the influence of exposure and outcomes aligned with the same effector allele. Additionally, palindromic SNPs were removed during the process.

### 2.5 Statistical analysis

#### 2.5.1 MR analysis

Based on the summarized data from 731 immunological traits (*n* = 3,757) and NAFLD (*n* = 778,614) derived from GWAS, the analysis was done in R software 4.3.2 utilizing the “TwoSampleMR” package 0.5.8 (available at http://www.Rproject.org). Inverse-variance weighted (IVW), weighted mode, weighted median, MR-Egger, and simple mode were adopted to illustrate the causal association between 731 immune traits and NAFLD, with IVW as the primary method [26,27]. Findings were visualized utilizing scatter, forest, and funnel plots. Given the risk of type 1 errors in multiple testing, the false discovery rate (FDR) correction was implemented.

### 2.6 Sensitivity analysis

Horizontal pleiotropy was checked by the MR-Egger method and MR-PRESSO tests [28], with *P* > 0.05 indicating that the IVs of immune cells did not have significant horizontal pleiotropy for NAFLD. Cochran’s *Q* statistics was used to judge heterogeneity in both the IVW and MR-Egger methods [29], with *P* > 0.05 implying no significant heterogeneity. The robustness of results was testified via the leave-one-out method.


**Informed consent:** Not applicable.
**Ethics approval:** Not applicable.

## 3 Results

### 3.1 Causal effect of immunophenotypes on NAFLD

**Figure 2:**
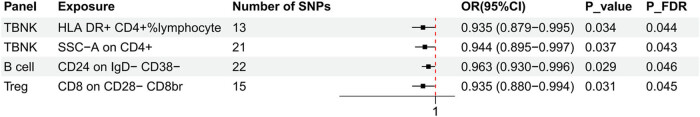
Forest plots showed protective effects of immunophenotypes (study group *n* = 3,757) on NAFLD (study group *n* = 778,614). TBNK, T cells, B cells, Natural killer cells; br, bright; HLA, Human Leucocyte Antigen.

**Figure 3:**
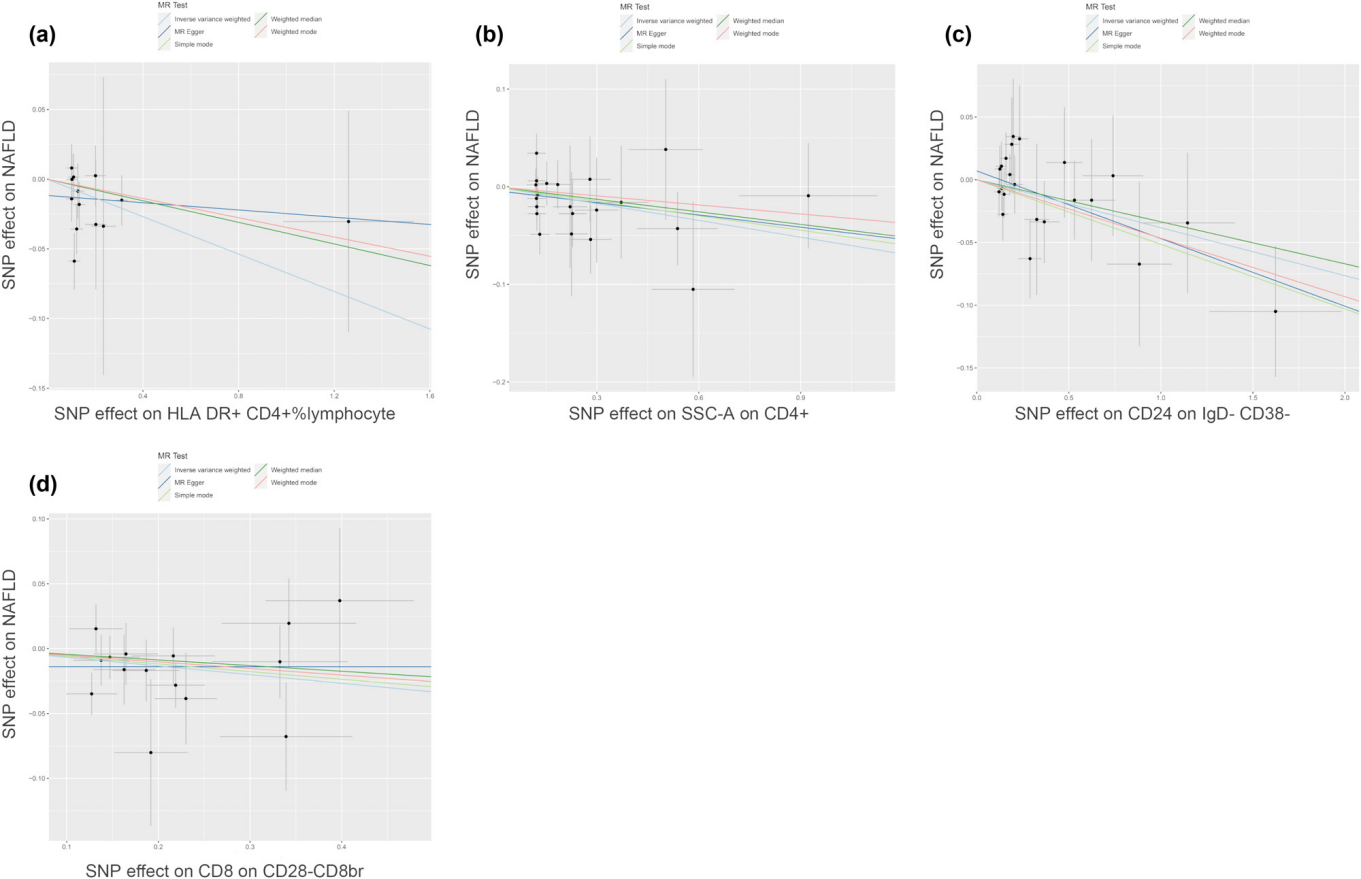
Causal effects of immune cells (study group *n* = 3,757) on NAFLD (study group *n* = 778,614). (a) Scatter plot between HLA-DR^+^ CD4^+^ % lymphocyte and NAFLD risk. (b) Scatter plot between SSC-A on CD4^+^ and NAFLD risk. (c) Scatter plot between CD24 on IgD^−^CD38^−^ and NAFLD risk. (d) Scatter plot between CD8 on CD28^−^CD8^br^ and NAFLD risk.

**Figure 4:**
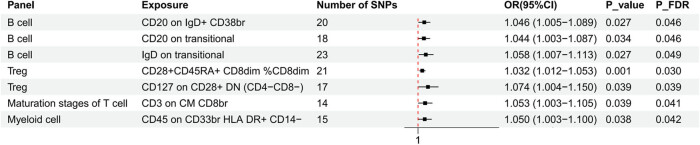
Forest plots showed promotional effects of immunophenotypes (study group *n* = 3,757) on NAFLD (study group *n* = 778,614). TBNK, T cells, B cells, Natural killer cells; DN, double negative; br, bright; CM, central memory; HLA, Human Leucocyte Antigen.

**Figure 5:**
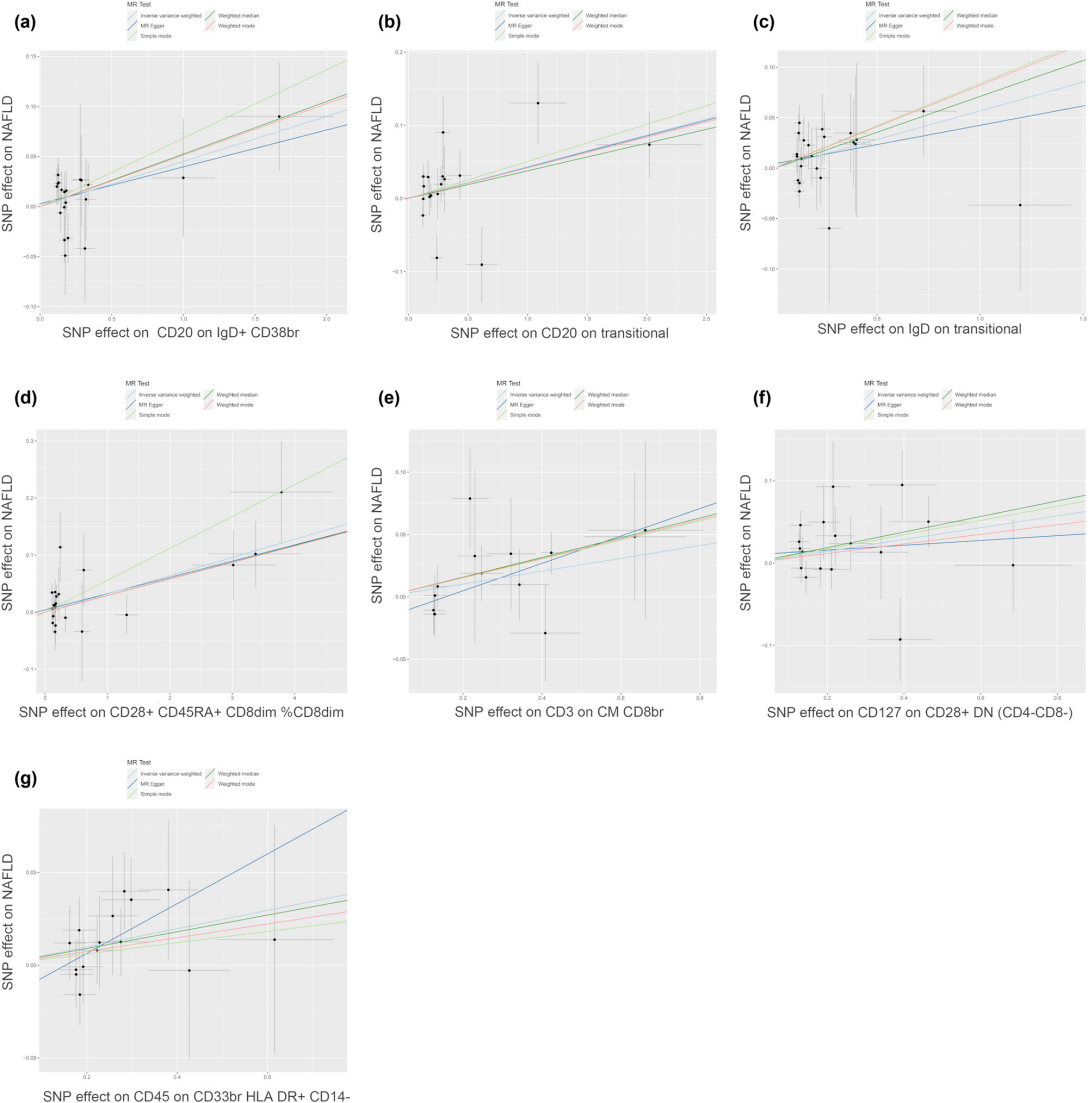
Causal effects of immune cells (study group *n* = 3,757) on NAFLD (study group *n* = 778,614). (a) Scatter plot between CD20 on IgD^+^ CD38^br^ and NAFLD risk. (b) Scatter plot between CD20 on transitional and NAFLD risk. (c) Scatter plot between IgD on transitional and NAFLD risk. (d) Scatter plot between CD28^+^ CD45RA^+^ CD8^dim^%CD8^dim^ and NAFLD risk. (e) Scatter plot between CD3 on CM CD8^br^ and NAFLD risk. (f) Scatter plot between CD127 on CD28^+^ DN (CD4^−^CD8^−^) and NAFLD risk. (g) Scatter plot between CD45 on CD33^br^ HLA-DR^+^ CD14^−^ and NAFLD risk.

MR analysis, primarily based on the IVW method, following FDR correction (*P*_FDR_ < 0.05), identified 11 immunophenotypes with causal associations with NAFLD. The comprehensive characteristics of the 11 immunophenotypes, including their parental populations, are shown in Table 1. HLA-DR^+^ CD4^+^ % lymphocytes (OR = 0.935, 95% CI = 0.879–0.995, *P* = 0.034, *P*_FDR_ = 0.044), SSC-A on CD4^+^ (OR = 0.944, 95% CI = 0.895–0.997, *P* = 0.037, *P*_FDR_ = 0.043), CD24 on IgD^−^CD38^−^ (OR = 0.963, 95% CI = 0.930–0.996, *P* = 0.029, *P*_FDR_ = 0.046), and CD8 on CD28^−^CD8^br^ (OR = 0.935, 95% CI = 0.880–0.994, *P* = 0.031, *P*_FDR_ = 0.045) showed a negative causal association with NAFLD (Figure 2). The scatter plots depicted a negative slope for these four immunophenotypes, indicating that the increase in the expression of these four immunophenotypes may decrease the likelihood of NAFLD (Figure 3). In contrast, CD28^+^ CD45RA^+^ CD8^dim^%CD8^dim^ (OR = 1.032, 95% CI = 1.012–1.053, *P* = 0.001, *P*_FDR_ = 0.003), CD127 on CD28^+^ DN (CD4^−^CD8^−^) (OR = 1.074, 95% CI = 1.004–1.150, *P* = 0.039, *P*_FDR_ = 0.039), CD20 on IgD^+^ CD38^br^ (OR = 1.046, 95% CI = 1.005–1.089, *P* = 0.027, *P*_FDR_ = 0.046), CD20 on transitional (OR = 1.044, 95% CI = 1.003–1.087, *P* = 0.034, *P*_FDR_ = 0.046), IgD on transitional (OR = 1.058, 95% CI = 1.007–1.113, *P* = 0.027, *P*_FDR_ = 0.049), CD3 on central memory (CM) CD8^br^ (OR = 1.053, 95% CI = 1.003–1.105, *P* = 0.039, *P*_FDR_ = 0.041), and CD45 on CD33^br^ HLA-DR^+^ CD14^−^ (OR = 1.050, 95% CI = 1.003–1.100, *P* = 0.038, *P*_FDR_ = 0.042) showed positive causal association with NAFLD (Figure 4). The scatter plots illustrated the positive slope of these seven immunophenotypes, indicating that as the expression of these seven immunophenotypes increased, the likelihood of NAFLD correspondingly enhanced (Figure 5).

**Table 1:** Comprehensive characteristics of the 11 immunophenotypes

Panel	GWAS ID	Trait	Parental population	Sample size	Number of SNPs	Trait type
TBNK	ebi-a-GCST90001626	HLA DR^+^ CD4^+^ %lymphocyte	CD45^+^ CD3^+^ CD4^+^	3,595	15,160,296	Relative count
TBNK	ebi-a-GCST90002081	SSC-A on CD4^+^	CD45^+^ CD3^+^ CD4^+^	3,113	14,903,739	MP
B cell	ebi-a-GCST90001769	CD24 on IgD^−^CD38^−^	CD19^+^IgD^−^CD38^−^	3,648	15,044,894	MFI
B cell	ebi-a-GCST90001751	CD20 on IgD^+^CD38^br^	CD19^+^ IgD^+^ CD38^br^	3,657	15,048,951	MFI
B cell	ebi-a-GCST90001763	CD20 on transitional	CD19^+^ CD38^+^ CD24^+^	3,657	15,048,951	MFI
B cell	ebi-a-GCST90001828	IgD on transitional	CD19^+^ CD38^+^ CD24^+^	3,657	15,048,951	MFI
Treg	ebi-a-GCST90002120	CD8 on CD28^−^CD8^br^	CD8^br^ CD28^−^	2,920	14,849,646	MFI
Treg	ebi-a-GCST90001665	CD28^+^ CD45RA^+^CD8^dim^%CD8^dim^	CD4^−^ CD8^dim^ CD28^+^ CD45RA^+^	3,440	15,147,619	Relative count
Treg	ebi-a-GCST90001925	CD127 on CD28^+^ DN (CD4^−^CD8^−^)	CD4^−^ CD8^−^ CD28^+^	2,918	14,849,609	MFI
Maturation stages of T cell	ebi-a-GCST90001846	CD3 on CM CD8^br^	CD4^−^ CD8^br^ CD45RA^−^ CCR7^+^	2,910	14,842,706	MFI
Myeloid cell	ebi-a-GCST90002042	CD45 on CD33^br^ HLA-DR^+^CD14^−^	CD45^+^ 7ADD^−^ CD14^−^ CD33^br^ HLA DR^+^	1,579	14,129,845	MFI

In addition to the IVW method, MR Egger (OR = 1.029, 95% CI = 1.006–1.052, *P* = 0.020), weighted median (OR = 1.030, 95% CI = 1.002–1.058, *P* = 0.036), simple mode (OR = 1.058, 95% CI = 1.004–1.114, *P* = 0.049), and weighted mode (OR = 1.029, 95% CI = 1.007–1.052, *P* = 0.016) yielded similar results on CD28^+^ CD45RA^+^ CD8^dim^%CD8^dim^. MR Egger (OR = 1.117, 95% CI = 1.016–1.226, *P* = 0.041), weighted median (OR = 1.082, 95% CI = 1.015–1.154, *P* = 0.015), and weighted mode (OR = 1.080, 95% CI = 1.011–1.155, *P* = 0.041) yielded similar results on CD3 on CM CD8^br^. MR Egger (OR = 0.948, 95% CI = 0.910–1.002, *P* = 0.044) yielded similar results on CD24 on IgD^−^CD38^−^. Weighted median (OR = 1.099, 95% CI = 1.008–1.198, *P* = 0.044) yielded similar results on CD127 on CD28^+^ DN (CD4^−^CD8^−^) (Table S1).

Forest plots showed SNP effects on the causal connections of immunophenotypes with NAFLD (Figure S1). In funnel plots, IVs were symmetrically distributed, which proved that the analysis followed the randomization principle (Figure S2).

### 3.2 Sensitivity analysis

Our findings indicated that all *Q*-pval values from the heterogeneity tests were >0.05, suggesting no significant heterogeneity. Additionally, MR-Egger and MR-PRESSO analyses demonstrated no significant horizontal pleiotropy for each immunophenotype, indicating that SNPs had no substantial impact on the outcome via exposure-unrelated factors (Table 2). The Leave-one-out plots had no significant biases, further proving the stability and reliability of the results (Figure S3).

**Table 2:** Tests for pleiotropy and heterogeneity between immune cells and NAFLD

Panel	Exposure	SNPs	MR Presso global test	MR_Egger regression		Heterogeneity		
			Pval	Intercept	P_intercept	Method	*Q*	*Q*-Pval
TBNK	HLA DR^+^ CD4^+^ %lymphocyte	13	0.57	−0.01	0.27	MR Egger	10.40	0.49
						IVW	11.78	0.46
	SSC-A on CD4^+^	21	0.74	0.00	0.70	MR Egger	15.76	0.67
						IVW	15.92	0.72
Treg	CD8 on CD28^−^ CD8br	15	0.68	−0.01	0.46	MR Egger	10.67	0.64
						IVW	11.26	0.67
	CD28^+^CD45RA^+^ CD8dim%CD8dim	21	0.60	0.00	0.52	MR Egger	18.95	0.46
						IVW	19.38	0.50
	CD127 on CD28^+^ DN (CD4^−^CD8^−^)	17	0.16	0.01	0.52	MR Egger	21.53	0.12
						IVW	22.15	0.14
B cells	CD24 on IgD^−^CD38^−^	22	0.94	0.01	0.39	MR Egger	11.27	0.94
						IVW	12.03	0.94
	CD20 on IgD^+^ CD38br	20	0.80	0.00	0.72	MR Egger	15.13	0.65
						IVW	15.26	0.71
	CD20 on transitional	18	0.15	0.00	0.96	MR Egger	25.78	0.06
						IVW	25.78	0.08
	IgD on transitional	23	0.65	0.00	0.64	MR Egger	18.87	0.59
						IVW	19.09	0.64
Maturation stages of T cells	CD3 on CM CD8br	14	0.77	−0.02	0.18	MR Egger	7.41	0.83
						IVW	9.44	0.74
Myeloid cells	CD45 on CD33br HLA DR^+^ CD14^−^	15	0.94	−0.02	0.31	MR Egger	6.14	0.94
						IVW	7.26	0.92

### 3.3 Causal effect of NAFLD on immunophenotypes

The IVW method was the primary method of reverse MR analysis on the 11 immunophenotypes. No causal association was revealed between NAFLD and any immunophenotype (Table S2 and Figure S4).

## 4 Discussion

The links between immune cells and NAFLD and the impact of genetics on NAFLD progression are not well understood [30]. Hence, the MR techniques were used to determine the possible causal association between 731 immune traits and NAFLD utilizing public genetic information. After effective screening, 199 SNPs associated with 11 immunophenotypes and NAFLD were screened as IVs. Our findings indicated that four immunophenotypes decreased the risk of NAFLD, including HLA-DR^+^ CD4^+^ % lymphocytes and SSC-A on CD4^+^ in the TBNK panel, CD24 on IgD^−^CD38^−^ in the B-cell lineage, and CD8 on CD28^−^ CD8^br^ in the Treg panel. In contrast, CD127 on CD28^+^ DN and CD28^+^ CD45RA^+^ CD8^dim^%CD8^dim^ in the Treg lineage, CD20 on IgD^+^ CD38^br^, CD20 on transitional, IgD on transitional in the B-cell panel, CD3 on CM CD8^br^ in the matured T-cell panels, and CD45 on CD33^br^HLA-DR^+^ CD14^−^ in the myeloid cell panel promoted NAFLD development. With IVW as the key method, analyses utilizing weighted median, MR Egger, and simple mode also yielded consistent results with those obtained from IVW in certain immune cell characteristics. This further strengthens our conclusions and enhances the reliability of the results.

T and B lymphocytes, representatives of adaptive immunity, demonstrate crucial roles in regulating immune responses and inflammation. T cells are grouped into CD4^+^, CD8^+^, and Treg cells. Our findings discovered that CD3 on CM CD8^br^ promoted NAFLD development. Consistent with the recent literature reports, the frequency of CM CD8^+^ T cells in human peripheral blood is positively associated with hepatic steatosis and lobular inflammation [31]. In addition, the frequency of CM CD8^+^ T cells is also significantly increased in the liver of NAFLD mouse models [32]. However, their precise mechanisms on NAFLD progression require further investigations.

Various preclinical and clinical studies have demonstrated that CD4^+^ T cells also contribute to NASH progression [33,34]. CD4^+^ T cell depletion using therapeutic antibodies could decrease the production of inflammatory cytokines and fibrosis, underscoring their significance in the clinical progression of NASH [35]. In contrast, we identified two CD4^+^ T cell subsets that exhibited protective effects against NAFLD:HLA-DR^+^ CD4^+^ lymphocytes and SSC-A^+^ CD4^+^ T cells. Recent articles have revealed the heterogeneity of CD4^+^ T cells [36]. Experimental evidence suggests that antigen-presenting cells (APCs) expressing Notch ligands can induce developing CD4^+^ T cells to express the anti-inflammatory cytokine interleukin (IL)-10, thereby exerting an opposite effect to typical CD4^+^ T cells [37]. IL-10 is vital in negatively regulating inflammation, mainly by selectively blocking inflammatory cytokines, cell-surface molecules, chemokines, and other molecules involved in inflammation [38]. Recent findings uncover that a newly discovered CD4^+^ T cell subset attenuates palmitate-induced lipotoxicity in the absence of IL-17 in a PI3K/AKT-dependent fashion [39,40]. This further highlights the heterogeneity of CD4^+^ T cells and the distinct functions of various T cell subsets. CD4^+^ T cell subsets identified in our research should be validated through further investigations.

Our research revealed that CD8 on CD28^−^CD8^br^ Treg cells exerted protective effects against NAFLD. They belong to CD8^+^ suppressor T cells [41] and are involved in the development of autoimmune diseases and immune tolerance in organ transplantation [42,43]. On the one hand, CD8^+^ CD28^−^ Treg cells upregulate ILT3 and ILT4 on DCs and monocytes, making these APCs tolerogenic cells, exhibiting low levels of costimulatory molecules and antigen-specific non-responsiveness in CD4^+^ T helper cells [44]. CD8^+^ CD28^−^ Treg cells are activated by the TLR2 pathway in macrophages predominantly via the production of IL-4 and IL-10, which are critical in preventing inflammatory responses [45]. These mechanisms all support the potential protection of CD8^+^ CD28^−^ Treg cells on NAFLD. CD127, also known as the IL-7R α chain, regulates the expression of recombination activating genes in double-negative T cells (DNTs) and initiates the VDJ rearrangement of the TCRβ chain, thus promoting the survival and proliferation of DNTs [46]. DNTs can activate the NLRP3 and TNFR2-STAT5-NF-κB signaling pathway by secreting TNF-α, thereby facilitating the differentiation of Th9 cells and contributing to liver fibrosis [47]. Earlier research has indicated that a subset of cytotoxic/inhibitory lymphocytes, characterized by CD3^+^ CD4^−^CD8^dim^, exhibits high expression of CD45RA in the peripheral blood lymphocytes of healthy individuals [48]. In comparison to CD8^dim^ T cells expressing CD45RO, these cells expressing CD45RA are in a naive state. Another study indicates that CD8^dim^ T cells with migratory capacity express high levels of CD28 [49]. CD28 is an important co-stimulatory molecule for T cells that plays a critical role in inflammatory diseases by upregulating inflammatory cytokines [50]. Therefore, we speculate that CD28^+^ CD45RA^+^ CD8^dim^ T cells may be a subset of naive CD8^+^ T cells with strong proliferative, activation, and migratory capacities. However, the relationship between CD28^+^ CD45RA^+^ CD8^dim^ T cells and NAFLD still requires further research for confirmation.

Our research indicated that four distinct types of B cells were associated with NAFLD progression. Previous investigations have shown the complex involvement of B cells in NAFLD progression due to the diverse B cell subtypes and their activities [51]. On the one hand, CD24 is heavily glycosylated and localized to lipid rafts on the B cell surface [52]. It is an initial protein expressed during the maturation of B cells in the late pre-B cell stage, like marginal B cells [53]. It can modulate immune functions by secreting IL-10 [54], thereby hindering NAFLD progression. On the other hand, the function of CD24 varies among B-cell subtypes and is linked to energy metabolism during B-cell differentiation [55]. Investigations have revealed that intrahepatic B cells are activated in mouse models of NASH, and NASH progression in mice can be markedly ameliorated through B-cell deficiency [56]. CD20, a surface protein specific to B cells, is the target of anti-CD20 antibodies in therapies for depleting B cells [57]. By targeting B cells, anti-CD20 monoclonal antibody therapy reduces inflammatory activity. Although the precise mechanism remains uncertain, this therapy can clinically relieve multiple diseases, such as multiple sclerosis and asthma [58,59]. This is in line with our findings and could offer a target for NAFLD treatment.

DCs originating from the myeloid lineage are also known as conventional dendritic cells (cDCs), which are integral components of the innate immune system and play a crucial role in both innate and adaptive immune responses [60]. Our research identified an immunophenotype characterized by CD45 on CD33^br^HLA-DR^+^ CD14^−^, which is derived from myeloid cells and may be a phenotype of cDCs that promotes NAFLD development. In patients with NAFLD/NASH, cDC1s are more abundant and activated, critically driving liver pathology by promoting the reprogramming of inflammatory T cells [61]. However, in mouse models, CD103 cDC1s have been identified as a protective subset of DCs that modulate the balance of proinflammatory and anti-inflammatory and protect the liver from metabolic injury [62]. cDC2s act as potent stimulators of CD4^+^ T cells, leading to the differentiation of helper T cells and guiding the immune system toward different pathways [63]. However, research on its relationship associated with NAFLD is currently limited. Further study is required to uncover the role of DCs in the development of NAFLD.

Nevertheless, the reverse MR analysis revealed that NAFLD did not appear to have a causal effect on immunophenotypes. However, as normal-NAFL-NASH progresses, immune-activated cell infiltration is significantly increased, indicating the remodeling of the immune microenvironment alongside disease progression [64]. The accumulation of liver metabolites due to NAFLD may lead to immune dysregulation. For instance, the depletion of fatty acid-induced cytotoxic CD4^+^ and self-reactive CXCR6^+^ CD8^+^ T cells, both essential for immune surveillance, could potentially initiate NAFLD and HCC progression [65]. Additionally, a recent study detected the distinct immunophenotypes and functions at different stages of NAFLD through cytometry by time-of-flight and bioinformatic analysis and revealed that the disease stages were associated with an inactive phenotype compared to controls [66]. Therefore, further foundational and clinical studies are warranted to establish the causal relationship between NAFLD and immunophenotypes.

This study, by MR analysis, illustrated the association between immune cells and NAFLD using data from a well-powered GWAS cohort. The merits of this investigation are highlighted as follows. First, the results were not disrupted by horizontal pleiotropy and confounders, preventing the likelihood of reverse causality. Second, the causal association between certain immunophenotypes and NAFLD was elucidated, paving the way for new immune targets in NAFLD treatment and providing a crucial theoretical basis for developing immunotherapeutic targets. Furthermore, an FDR was utilized to address statistical biases from multiple comparisons and to control false positives in multiple hypothesis testing.

## 5 Limitation

Nevertheless, our research also has constraints. First, our research relied on a European database, which might introduce demographic bias into the MR findings. Subsequent studies should incorporate various ethnic backgrounds while also segmenting data by gender and other demographic factors. Second, the results were analyzed using a relaxed threshold, which could result in some false positives, although it facilitated a more detailed exploration of the pronounced link between immune cells. Moreover, confounders could not be ruled out completely, although sensitivity analysis was performed to exclude SNPs associated with potential confounders. Further investigation is necessary to uncover the complex connection between diverse innate and adaptive immune cells and NAFLD and to delineate their precise mechanisms.

## 6 Conclusion

The MR analysis reveals a potential genetic link between immunophenotypes and NAFLD. Furthermore, our results refine the theoretical understanding of NAFLD-immune crosstalk, providing a fresh framework for immunoregulation in NAFLD therapy.

## Abbreviations

AC: absolute cell counts
APCs: antigen presenting cells
CM: central memory
DAMPs: damage-associated molecular patterns
DCs: dendritic cells
FDR: false discovery rate
GWAS: genome-wide association study
HCC: hepatocellular carcinoma
IV: instrumental variable
IVW: inverse-variance weighted
MFI: median fluorescence intensity
MP: morphological parameters
MR: Mendelian randomization
NAFLD: non-alcoholic fatty liver disease
NASH: non-alcoholic steatohepatitis
PRRs: pattern recognition receptors
RC: relative cell counts
SNPs: single-nucleotide polymorphisms
TBNK: T cells, B cells, and natural killer cells

## Supplementary Material

Supplementary Figure

Supplementary Table
