# Supplementary material for: Examination of the causal role of immune cells in non-alcoholic fatty liver disease by a bidirectional Mendelian randomization study
Source: Open Med (Wars). 2025 Feb 19;20(1):20251154. doi: 10.1515/med-2025-1154 (PMC11843165; doi:10.1515/med-2025-1154)
Supplement: Supplementary Figure [file med-2025-1154-sm1.pdf]

Supplementary material

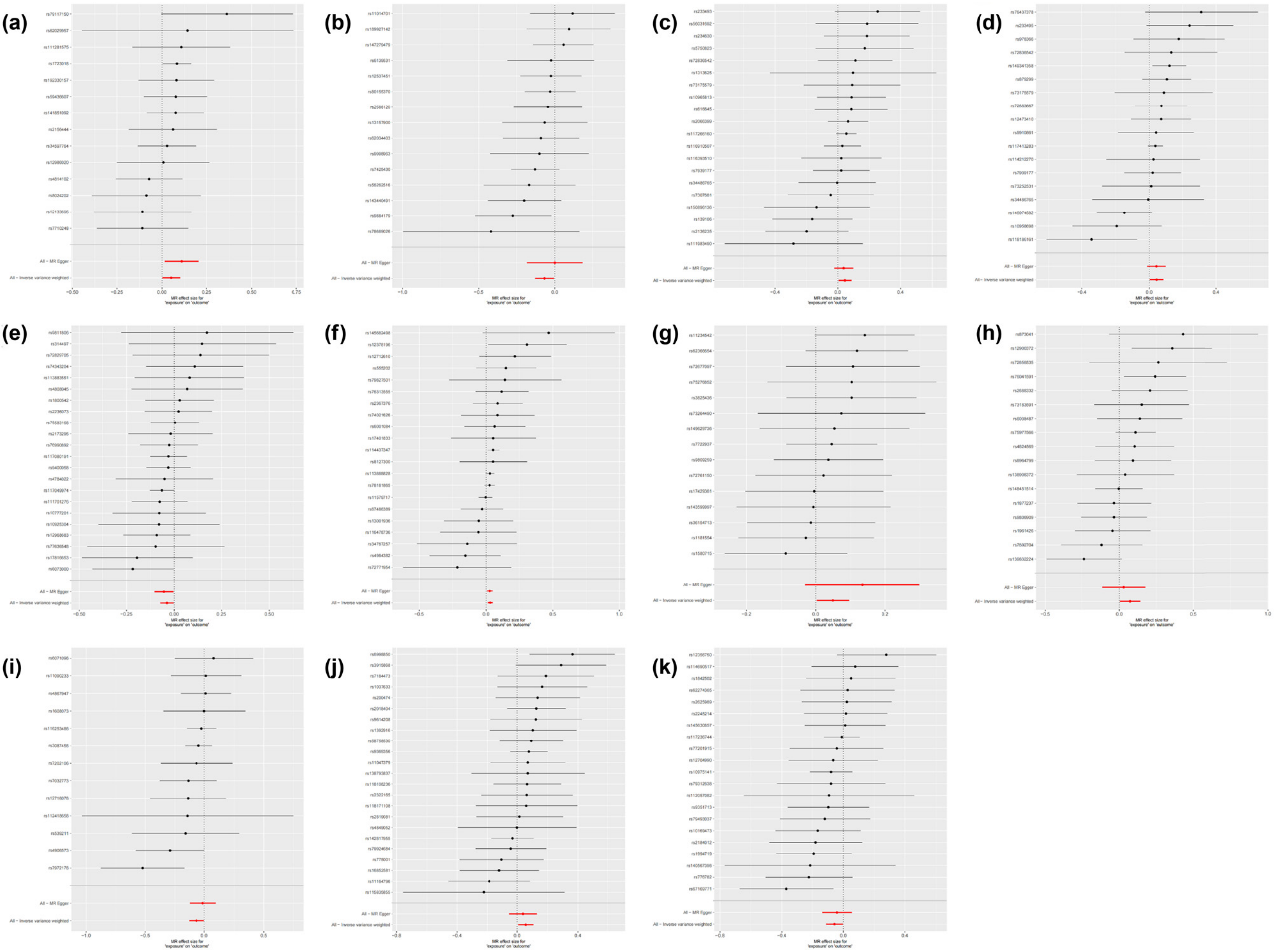

Figure S1: Forest plots between immune cells on NAFLD. (a) Forest plot between CD3 on CM CD8br and NAFLD risk; (b) Forest plot between CD8 on CD28- CD8br and NAFLD risk; (c) Forest plot between CD20 on IgD+ CD38br and NAFLD risk; (d) Forest plot between CD20 on transitional and NAFLD risk; (e) Forest plot between CD24 on IgD- CD38- and NAFLD risk; (f) Forest plot between CD28+ CD45RA+ CD8dim %CD8dim and NAFLD risk; (g) Forest plot between CD45 on CD33br HLA DR+ CD14- and NAFLD risk; (h) Forest plot between CD127 on CD28+ DN (CD4-CD8-) and NAFLD risk; (i) Forest plot between HLA DR+ CD4+ %lymphocyte and NAFLD risk; (j) Forest plot between IgD on transitional and NAFLD risk; (k) Forest plot between SSC-A on CD4+ and NAFLD risk.

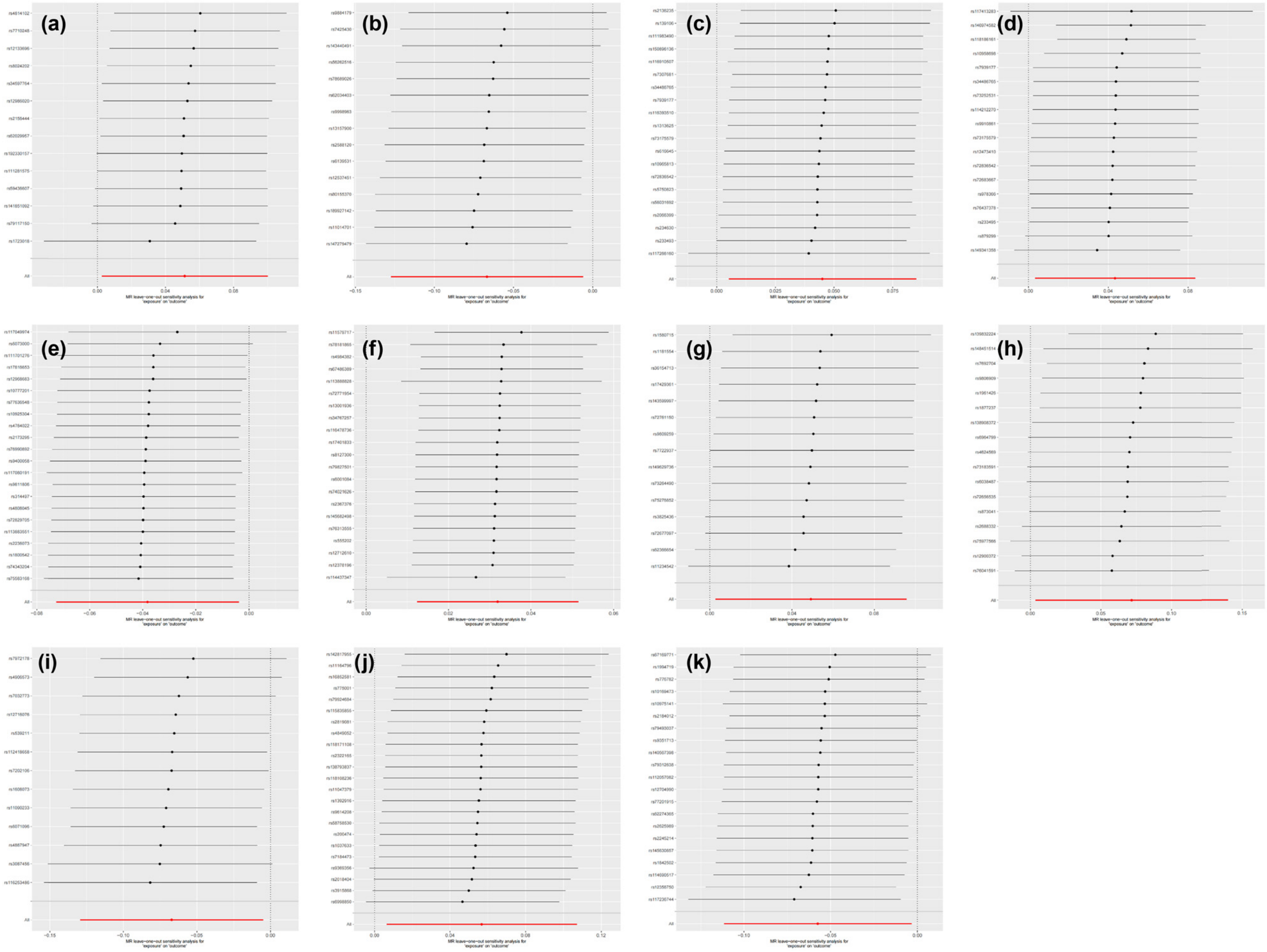

Figure S2: Leave-one-out plots for the causal association between immune cells and NAFLD. (a) Leave-one-out plot between CD3 on CM CD8br and NAFLD risk; (b) Leave-one-out plot between CD8 on CD28- CD8br and NAFLD risk; (c) Leave-one-out plot between CD20 on IgD+ CD38br and NAFLD risk; (d) Leave-one-out plot between CD20 on transitional and NAFLD risk; (e) Leave-one-out plot between CD24 on IgD- CD38- and NAFLD risk; (f) Leave-one-out plot between CD28+ CD45RA+ CD8dim %CD8dim and NAFLD risk; (g) Leave-one-out plot between CD45 on CD33br HLA DR+ CD14- and NAFLD risk; (h) Leave-one-out plot between CD127 on CD28+ DN (CD4-CD8-) and NAFLD risk; (i) Leave-one-out plot between HLA DR+ CD4+ % lymphocyte and NAFLD risk; (j) Leave-one-out plot between IgD on transitional and NAFLD risk; (k) Leave-one-out plot between SSC-A on CD4+ and NAFLD risk.

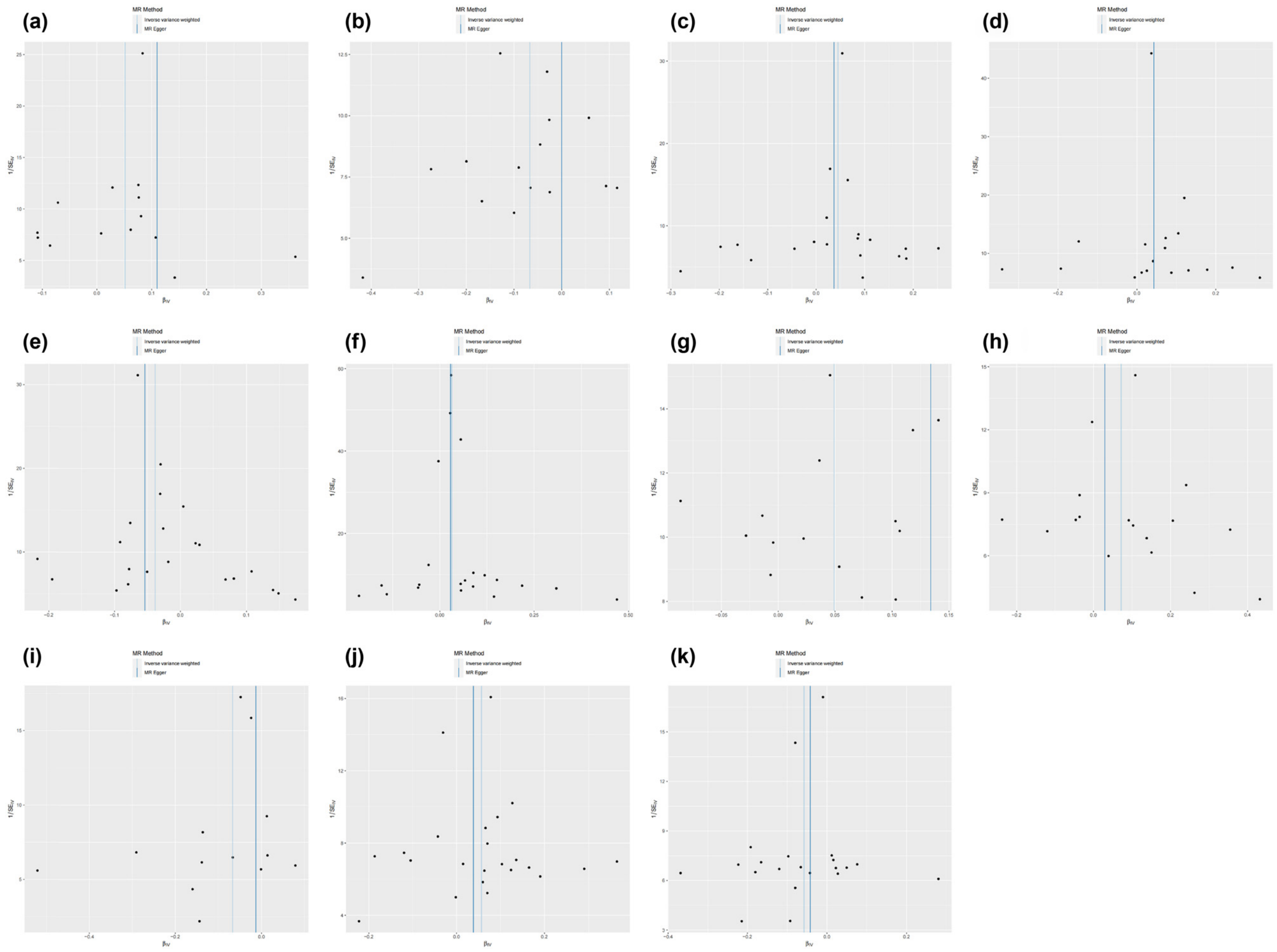

Figure S3: Funnel plots for the causal association between immune cells and NAFLD. (a) Funnel plot between CD3 on CM CD8br and NAFLD risk; (b) Funnel plot between CD8 on CD28- CD8br and NAFLD risk; (c) Funnel plot between CD20 on IgD+ CD38br and NAFLD risk; (d) Funnel plot between CD20 on transitional and NAFLD risk; (e) Funnel plot between CD24 on IgD- CD38- and NAFLD risk; (f) Funnel plot between CD28+ CD45RA+ CD8dim %CD8dim and NAFLD risk; (g) Funnel plot between CD45 on CD33br HLA DR+ CD14- and NAFLD risk; (h) Funnel plot between CD127 on CD28+ DN (CD4-CD8-) and NAFLD risk; (i) Funnel plot between HLA DR+ CD4+ %lymphocyte and NAFLD risk; (j) Funnel plot between IgD on transitional and NAFLD risk; (k) Funnel plot between SSC-A on CD4+ and NAFLD risk.

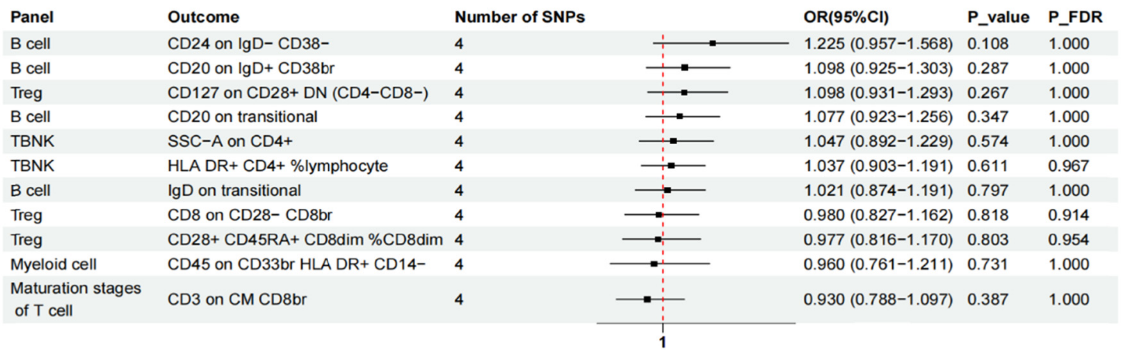

Figure S4: Forest plot showed the causal relationship between NAFLD and immunophenotypes.
